# A huge malignant solitary fibrous tumor of kidney: case report and review of the literature

**DOI:** 10.1186/1746-1596-9-13

**Published:** 2014-01-20

**Authors:** Hui Wang, Qing Liao, Xin Liao, Ge Wen, Zuguo Li, Chuang Lin, Liang Zhao

**Affiliations:** 1 Department of Medical Oncology, Affiliated Cancer Hospital of Guangzhou Medical University, Guangzhou, China; 2 Department of Pathology, School of Basic Medical Sciences, Southern Medical University, Guangzhou, China; 3 Department of Radiology, Nanfang Hospital, Southern Medical University, Guangzhou, China; 4 Department of Imaging Center, Nanfang Hospital, Southern Medical University, Guangzhou, China; 5 Department of Pathology, Nanfang Hospital, Southern Medical University, Guangzhou, China

## Abstract

**Virtual slides:**

The virtual slide(s) for this article can be found here: http://www.diagnosticpathology.diagnomx.eu/vs/1603694556107408.

Solitary fibrous tumor (SFT) is a spindle cell neoplasm that rarely occurs in the kidney. Malignant SFT of the kidney is particularly rare. Here, we report a 66-year old woman with a right flank mass that has been proved clinically and radiographically. Grossly, the largest diameter of the mass were measured up to 23 cm, was poorly circumscribed. Approximately 80% of the neoplasm consisted of hyperchromatic and pleomorphic spindled cells surrounding staghornlike blood vessels. Tumor cells frequently had mitoses and necrosis. However, the remainder of the mass was composed of haphazard, storiform or short fascicular arrangements of spindle cells in a loose myxoid to fibrous stroma. Immunohistochemically, we observed diffusely strong CD34 staining and an 85% Ki-67 proliferative index. The tumor partly showed negative CD34 and a 20% proliferative index. To our knowledge, this is the largest malignant renal SFT in the reported literatures and shows an obviously high proliferative index.

## Background

Solitary fibrous tumor (SFT) is uncommon type of neoplasm of mesenchymal origin, most frequently arising in the pleura. It rarely occurs in extrapleural sites like upper respiratory tract, lung, nasal cavity, paranasal sinuses, orbits, mediastinum, major salivary glands, breast, meninges, liver and urogenital organs [1,2]. Morphologically, SFT is characterized by spindle cell proliferation with a patternless architecture, and a final diagnosis is made only after immunohistochemical study [3]. SFTs arising in the kidney were first described in 1996 by Gelb *et al.*[4]; however, few cases, particularly those involving malignancy, have been reported in the worldwide literature to date [5]. We herein report what is, to our knowledge, the largest malignant renal SFT in a 66-year old woman; we discuss its clinical, light microscopic, and immunohistochemical features, and differential diagnosis.

## Case presentation

### Clinical summary

**Figure 1:**
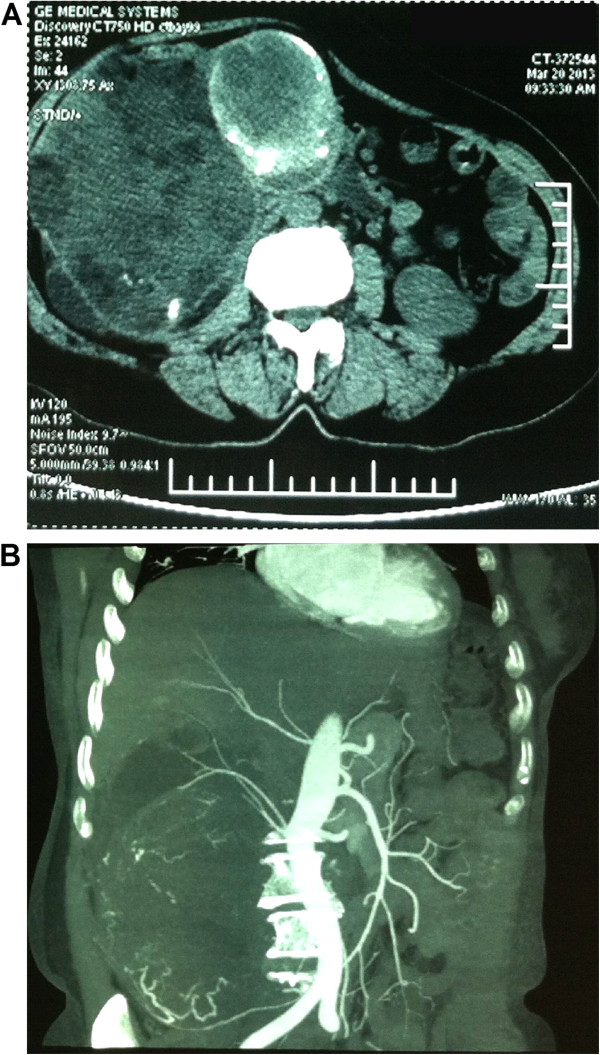
**Computed topography (CT) of the abdomen. A**, CT scan images showed a huge lobulated cystic-solid tumor occupying the perirenal space of right kidney. **B**, The coronal reconstruction image showed a shadows of multiple tortuous vessels at the margin of huge mass.

A 66-year old woman was admitted to our hospital with two-year history of a right flank mass, without other constitutional symptoms. The patient denied a history of unhealthful environment and asbestos exposure, malnutrition and treatment of additional diseases. Laboratory findings were unremarkable. Physical examination revealed a hard right abdominal mass. A subsequent computed topography (CT) of the abdomen showed a huge lobulated cystic-solid tumor occupying the perirenal space of right kidney without evidence of either local invasion or lymphadenopathy (Figure 1). The patient underwent right radical nephrectomy under a pre-operative diagnosis of American Joint Committee on Cancer (AJCC) stage II (T2aN0) renal cell carcinoma. Post-operation course were smooth. Neither chemotherapy nor radiation therapy was given. She has been well without evidence of recurrence or metastasis for nine months.

### Pathologic findings

**Figure 2:**
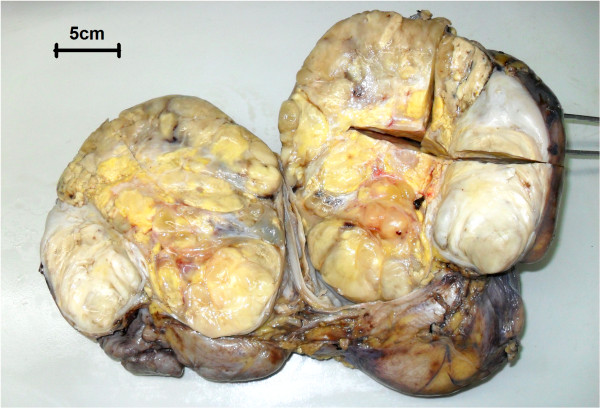
**Gross morphology.** The tumor was firm and showed a yellowish white to tran-gray, myxoid and lobulated cut surface with prominent hemorrhage and necrosis.

A nephrectomy specimen (26 × 20 × 12 cm) with attached ureter and perirenal fibroadipose tissue was received. The specimen was bisected to reveal a 23 × 18 × 12 cm irregular and unencapsulated tumor occupying the perirenal space of the upper and middle poles of kidney. The tumor was firm and showed a yellowish white to tran-gray, myxoid and lobulated cut surface with prominent hemorrhage and necrosis (Figure 2).

**Figure 3:**
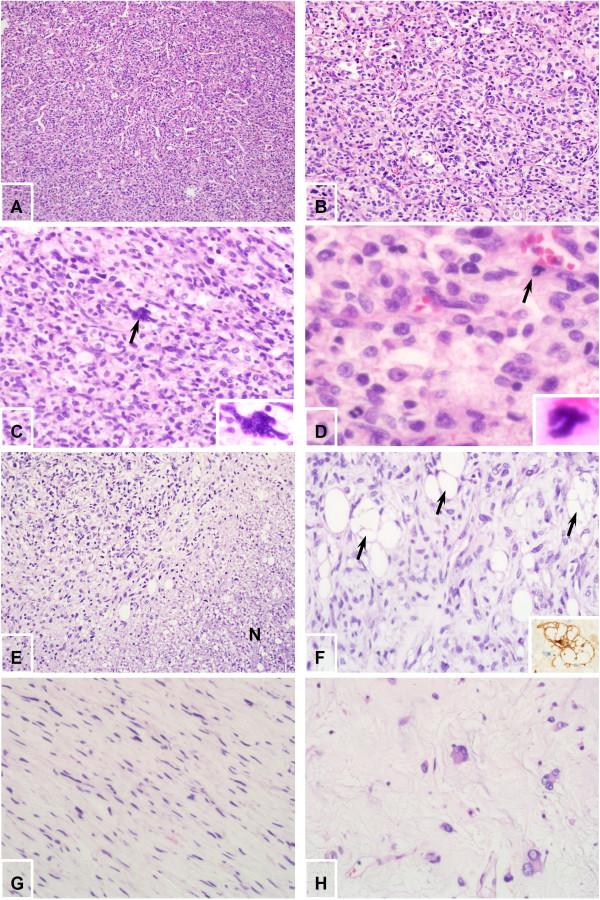
**Microscopic features. A** &**B**, Hyperchromatic and pleomorphic spindled cells surrounding staghornlike blood vessels (**A**,×100 original magnification; **B**, ×200 original magnification. **C**, Tumor giant cells (arrow, ×400 original magnification). **D**, Abnormal mitoses (arrow, ×1000 original magnification). **E**, Tumor necrosis (N) (×200 original magnification). **F**, sporadic mature adipose tissue (×400 original magnification). Adipose cells showed S-100 immunostaining (inset, ×400 original magnification). **G**, Storiform arrangements of spindle cells in fibrous stroma containing dense collagen fibers (×400 original magnification). **H**, Haphazard arrangements of multinucleated floret-like cells in a loose myxoid stroma (×400 original magnification).

Microscopic examination revealed two distinct histologic appearances. The first, a typical cellular area, accounted for more than 80% of the sampled tumor. The area consisted of hyperchromatic and pleomorphic spindled cells surrounding staghornlike blood vessels (Figure 3A, B). Tumor cells frequently had mitoses 6–8 per 10 high power fields. Tumor giant cells (Figure 3C) and abnormal mitoses (Figure 3D) were occasionally seen. Tumor necrosis were evidently present (Figure 3E). We also find sporadic mature adipose tissue (Figure 3F). The remaining 20% was characterized by haphazard, storiform or short fascicular arrangements of spindle cells in a loose myxoid to fibrous stroma containing dense collagen fibers (Figure 3G, H).

**Figure 4:**
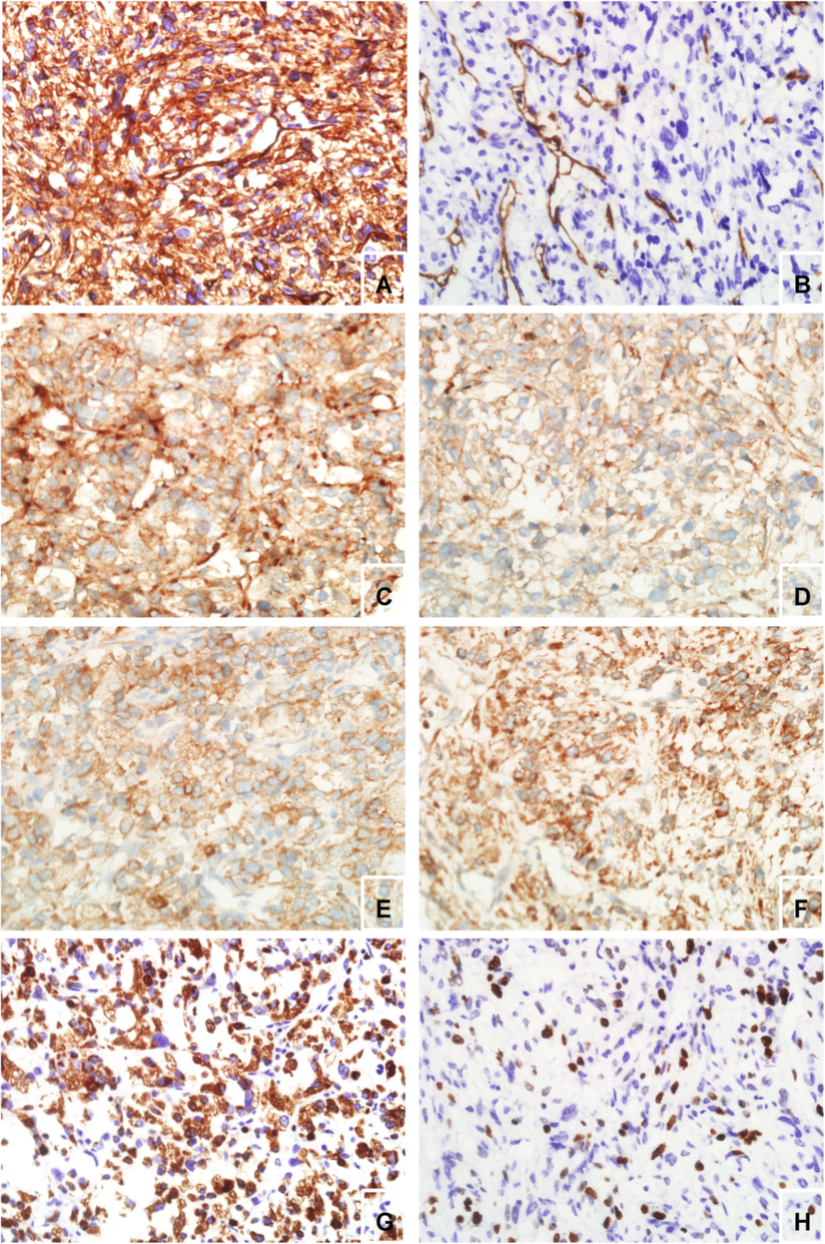
**Immunohistochemical staining.** Left panel, the lesion showed diffusely strong CD34 **(A)**, CD99 **(C)**, Bcl-2 **(E)** staining and an 85% Ki-67 proliferative index **(G)** (×200 original magnification). Right panel, the other lesion showed diffusely negative CD34 **(B)**, positive CD99 **(D)**, positive Bcl-2 **(F)** staining and a 20% Ki-67 proliferative index **(H)** (×200 original magnification).

Immunohistochemically, the tumor mostly showed diffusely strong CD34 (Figure 4A), CD99 (Figure 4C), Bcl-2 (Figure 4E) and vimentin staining. Ki-67 immunostaining showed an 85% proliferative index. However, the tumor partly showed negative CD34 and a 20% proliferative index. They totally stained negatively for S100, HMB45, actin, desmin, CD10, cytokeratin, cytokeratin 7, CD31, EMA, and CD68.

## Discussion

SFT was first reported by Klemperer and Rabin in 1931 as a tumor of the pleura [6]. SFT is a rare spindle cell neoplasm that usually arises in the pleura [7]. However, in recent years, there have been several reports of SFT arising in other organs, including the kidney [4,7]. Although the histogenesis of SFT remains undetermined, recent studies strongly favor a primitive mesenchymal or perivascular cell origin [8]. SFT reveals diffusely positive reactivity for CD34 which is now considered to be a characteristic marker of SFT [9]. Other positive markers found frequently in SFT include Bcl-2 and CD99 [10]. On the contrary, SFT in general shows negative reactivity for cytokeratin, alpha-SMA, S-100, CD31 and c-kit. Frequent expression of accessible ligands for endogenous lectins galectins-1 and -3, the expression of the angiogenic macrophage migration inhibitory factor (MIF), and the dense vascularization intimate a functional relationship [11]. These markers contribute to the differential diagnosis of SFT from other spindle cell tumors.

The majority of SFTs are benign; however, a few have revealed histologically malignant features. The criteria for clinical malignancy in intrathoracic SFT, first proposed by England *et al*. in 1989, include increased cellularity, pleomorphism, mitotic count more than 4 per 10 high power fields, necrosis, hemorrhage, size more than 10 cm, non-pedunculated and atypical locations (parietal pleura, pulamonary parenchyma) [12]. The diagnostic criteria for malignant extrathoracic SFTs are purely microscopic and include increased cellularity, pleomorphism and mitotic count more than 4 per 10 high power fields [13]. Malignant SFT of the kidneys is rare, and only 10 cases have been reported (Table 1). Fine *et al*. [14] reported the first case of malignant renal SFT to develop distant metastasis. Our case fulfilled the diagnostic criteria for malignant extrathoracic SFTs. Additionally, the presence of necrosis and a 85% Ki-67 proliferative index further supports a diagnosis of malignant renal SFT. We herein report what is, to our knowledge, the largest malignant renal SFT with a highest proliferative index.

**Table 1:** Clinicopathological findings of malignant solitary fibrous tumors of the kidney in the literature

**Case**	**Year**	**Age**	**Sex**	**Symptom**	**Side**	**Tumor size, cm**	**Treatment**	**Follow-up (months)**	**Outcome**	**Mitoses/10HPF**	**CD34**	**CD99**	**Bcl-2**	**Ki-67 (%)**
1 [15]	2013	49	F	Dyspnea and chest pain	L	NA	Nephrectomy and interferon	23	NED	NA	POS	POS	POS	NA
2 [16]	2012	72	M	Flank pain	L	20	Nephrectomy	33	Recurrence	2-5	POS	NEG	POS	2–7
3 [17]	2012	68	F	Flank pain	NA	7	Nephrectomy	5	Died of disease	NA	POS	POS	POS	NA
4 [18]	2012	60	M	Incidental	R	NA	Nephrectomy	5	Spinal metastasis	20	POS	POS	POS	25
5 [10]	2008	34	F	Flank pain	L	9	Nephrectomy	15	NED	2-6	POS	POS	POS	>10
6 [19]	2011	72	F	Abdominal discomfort	L	19	Nephrectomy	15	NED	>4	POS	POS	NEG	NA
7 [14]	2006	76	M	Abdominal mass	L	12	Nephrectomy	4	Lung metastasis	Frequent	POS	NEG	POS	NA
8 [20]	2011	50	F	Flank pain	R	9	Nephrectomy	30	NED	8	POS	POS	NEG	20
9 [21]	2009	72	F	Abdominal mass	L	19	Nephrectomy	NA	NA	Frequent	NA	NA	NA	NA
10 [22]	2012	56	M	Shortness of breath	L	10,10	Nephrectomy	10	NED	>4	POS	POS	NA	NA
11	Present	65	F	Abdominal mass	R	23	Nephrectomy	6	NED	6-8	POS	POS	POS	85

M = male; F = female; NA = not available; R = right; L = left; NED = no evidence of disease; POS = Positive; NEG = negative.

Malignant phenotypes can occur in 10% to 15% of intrathoracic SFTs and up to 10% of extrathoracic SFTs [23]. Malignant SFT is postulated to develop via two pathway: (1) *de novo* occurrence or (2) dedifferentiation or sarcomatous overgrowth from a pre-existing histologically benign SFT [1,20]. Margo *et al*. and Fine *et al*. have reported two cases of malignant renal SFTs developing via dedifferentiation or sarcomatous overgrowth from a pre-existing benign SFT [10,14]. Hsieh *et al*. showed a case of de novo malignant renal SFT without any areas of dedifferentiation [20]. The tumor reported by Fine *et al*. had infiltrative borders and focal necrosis, and invaded beyond the renal capsule. The tumor described by Margo *et al*. was a circumscribed mass devoid of either hemorrhage or necrosis with a 3-cm nodular area. In contrast, the tumors of Hsieh *et al*. and us showed an unencapsulated border with prominent necrosis and hemorrhage. Microscopically, the tumor reported by Fine *et al*. and Margo *et al*. showed typical features of benign SFT with 90% and 30% of dedifferentiation or sarcomatous overgrowth, respectively. In contrast, the tumors of Hsieh *et al*. and us appeared to develop de novo since we did not find any areas of dedifferentiation after extensive tumor sampling.

SFT shows a wide variety of microscopic growth patterns and should be distinguished from benign and malignant spindle cell tumors. Positive immunoreactivity for CD34 and CD99 is characteristic of SFT, and highly valuable in differentiating from other mesenchymal tumors. However the expression of CD34 and CD99 may be decreased or absent in areas with marked atypia or dedifferentiation [14,24]. in addition, CD34 expression was negative in the resected tissue from the liver and weakly positive in the resected tissue from the lungs. Thus, Hideo *et al*. postulated that the loss of CD34 expression might promote tumor metastasis tot other organs, and could lead to malignant transformation from the benign tumor relevant to fatal outcome. Similarly, our case of SFT showed negative CD34 and a lower proliferative index in part of the lesion. The CD34-negative part of the lesion was morphologically indistinguishable from its CD34-positive region, which may participate in malignant outcome. Further research is needed to clarify these points.

SFTs must be differentiated from the malignant and benign spindle cell tumors of the kidney. Its differentiation from other primary monomorphous benign and malignant spindle cell tumors of the kidney, such as fibroma, benign fibrous histiocytoma, hemangiopericytoma, inflammatory myofibroblastic (pseudo-)tumor, leiomyoma, angiomyolipoma with predominant spindle cell smooth muscle component, benign peripheral nerve sheath tumors, renal mixed epithelial/stromal tumors, adult type mesoblastic nephroma, fibrous type monophasic synovial sarcoma, malignant peripheral nerve sheath tumors, fibrosarcoma, and low-grade fibromyxoid sarcoma [25]. It is particularly difficult to differentiate it especially from hemangiopericytoma (HPC) that has similar histological findings and CD34 positivity. Nearly 30 cases of renal HPC have been reported in the literature. HPCs macroscopically contain hemorrhagic areas. Compared to SFTs, HPCs have less cellular diversity and stromal hyalinization. While CD34 positivity is weak in HPC, it is diffuse and strong in SFT. In conclusion, immunohistochemical examination is the main diagnostic method. SFTs generally have a favorable prognosis. A majority of them do not recur or metastasize. Malignant behaviors such as metastasis and recurrence are observed in 10% of extrapleural tumors [23]. Malignant behavior, however, has been reported in at least 4 of 10 cases in the literature (Table 1) [14,16-18]. A careful and adequate resection is the mainstay of therapy of renal SFT. The fole of adjuvant chemotherapy is still unclear. Cuello *et al*. have attempted to manage with subcutaneous interferon achieving 23 months of progression-free survival [15]. Given that it is difficult to predict the aggressive clinical behavior of the tumor, it is of paramount importance to follow the patients at a regular basis. We did not identify any recurrence or metastasis at the 6^th^ month of followpu following surgery in our patient.

### Consent

Written informed consent was obtained from the patient for the publication of this report and any accompanying images.

## Competing interests

The authors declare that they have no competing interests.

## Authors' contributions

HW, CL carried out the molecular experiments, GW, XL performed image observation, QL analyzed the data, ZGL, LZ participated in histological observation and LZ drafted the manuscript. All authors read and approved the final manuscript.
